# Tracing ultrahigh-pressure metamorphism at the catchment scale

**DOI:** 10.1038/s41598-018-21262-8

**Published:** 2018-02-13

**Authors:** Jan Schönig, Guido Meinhold, Hilmar von Eynatten, Nils K. Lünsdorf

**Affiliations:** 1 Department of Sedimentology and Environmental Geology, Geoscience Centre Göttingen, University of Göttingen, Goldschmidtstraße 3, 37077 Göttingen, Germany; 2 School of Geography, Geology and the Environment, Keele University, Keele, Staffordshire ST5 5BG UK

## Abstract

Finding traces of ultrahigh-pressure (UHP) metamorphism in the geological record has huge implications for unravelling Earth’s geodynamic evolution, such as the onset of deep subduction. Usually, UHP rocks are identified by specific mineral inclusions like coesite and characteristic petrographic features resulting from its (partial) transformation to the lower-pressure polymorph quartz in thin sections of crystalline rocks. This approach relies on very small sample size and is thus limited to a few points within large regions. Here we present the first findings of coesite inclusions in detrital mineral grains. The intact monomineralic inclusions were detected in garnets from a modern sand sample from the Western Gneiss Region, SW Norway. They represent the first known intact monomineralic coesite inclusions in the Western Gneiss Region, and their presence is suggested to indicate the erosion of UHP rocks in the sampled catchment area. The novel approach introduced here allows for tracing UHP metamorphic rocks and their erosional products at the catchment scale instead of being limited to outcrops of crystalline rocks. It opens new avenues for the prospective exploration of UHP metamorphism in Earth’s geological record.

## Introduction

The term ultrahigh-pressure (UHP) metamorphism refers to crustal rocks which experienced pressure–temperature (*P–T*) conditions high enough for the formation of coesite^1,2^, i.e., pressures of >2.6 GPa at 600 °C or >2.8 GPa at 900 °C^3–5^. Tracing UHP rocks and their corresponding terranes, which underwent these extreme conditions, determining their areal extent and geodynamic context, and dating these events has major implications for our understanding of the principal geodynamic processes that control the evolution of planet Earth in space and time. In particular, the detection of the oldest UHP rocks is of extraordinary interest because they are considered to mark the onset of deep (>100 km) subduction in the rock record, i.e., the transition to the modern plate tectonics regime^6^. So far, the oldest undoubted UHP terranes are of Neoproterozoic age^7–9^, which forms a central argument for the hypothesis that modern-style subduction tectonics had not taken place before^10,11^. However, first indications of conditions close to, or within, the *P–T* field of UHP metamorphism experienced by Paleoproterozoic crustal rocks are given by geobarometry, phase equilibria modelling, and exsolution textures^12–15^, but indisputable UHP mineral indicators (coesite or certain metamorphic microdiamond) are still lacking for rocks older than Neoproterozoic.

## Ultrahigh-pressure terranes and mineral indicators

The first UHP terranes were discovered in 1984^16,17^ and to date more than 30 occurrences are known^18^, indicating that UHP metamorphism is a common process in major orogenic cycles at least since the late Neoproterozoic^1,19^. Although several UHP indicator minerals were identified in crystalline rocks during the last three decades, still the most prominent indicators for the detection of UHP rocks are coesite and microdiamond. Particularly the presence of coesite (or pseudomorphs after coesite) is crucial in most cases because coesite is (i) the high-pressure polymorph of silica, which is abundant in most crustal rocks, and (ii) per definition present from the point where UHP conditions are reached. The most critical factor in the preservation of coesite is the availability of fluids during exhumation^20^. Unless fluids are almost absent along the retrograde path following UHP metamorphism^21^, intergranular coesite will be replaced by quartz when *P–T* conditions drop below the quartz/coesite equilibrium line. In contrast, inclusions of coesite enclosed in mechanically robust host minerals like zircon or garnet can persist up to surface conditions, because these host minerals sustain a certain inclusion overpressure compared to the external (lithostatic) pressure^16,22^. Consequently, the coesite inclusions are insulated from metamorphic fluids as long as the host phase is intact (i.e., no fracturing) and thus shielded from the coesite to quartz transformation. Often the inclusion overpressure exceeds the tensile strength of its host mineral at a specific point of the retrograde path, resulting in fracturing of the host, which connects the inclusion to the external conditions. This leads to a delayed transformation into quartz associated with a volume expansion of roughly 10%^1,22^, whereas temperature and fluid availability control the reaction kinetics^20,23^. The typical structures of the former monomineralic coesite inclusions are either bimineralic SiO_2_ inclusions with relictic coesite cores and fine-grained polycrystalline (palisade) quartz rims or (if the degree of transformation is higher) monomineralic polycrystalline quartz inclusions. Both types show radial expansion fractures originating from the inclusion/host boundary and spreading out into the host mineral^21^.

## Tracing ultrahigh-pressure terranes/rocks

When exploring a region regarding UHP metamorphic rocks, the general approach is to prepare thin sections from crystalline rocks, which have been sampled for the highest potential to be equilibrated under UHP conditions (mainly eclogites), and to seek for the typical structures of pseudomorphs after coesite under the polarisation microscope. Beyond obvious drawbacks due to the subjective selection of samples in the field, this method suffers especially from (i) sampling only very small points from potentially huge rock volumes, (ii) preparing just a few thin sections, which implies a high probability of missing the relevant structures, (iii) overlooking other UHP indicators (including monomineralic coesite), (iv) overlooking other potential rocks due to overprinting, and (v) being restricted to UHP rocks that still exist and are exposed at the Earth’s surface. The latter two are particularly unfavourable for the detection of old UHP terranes because the global metamorphic record is skewed by overprinting and erosion^15^. Of specific interest are also felsic rocks, which are often the dominant rock type in UHP terranes enveloping the mafic pods (eclogites), and are very likely to have undergone pervasive overprinting under lower pressure facies conditions during exhumation^18,24^. Some of these felsic rocks potentially also crystallised under UHP conditions, but they are rarely sampled and analysed regarding UHP metamorphism because relicts of peak metamorphism are difficult to find by point sampling.

**Figure 1:**
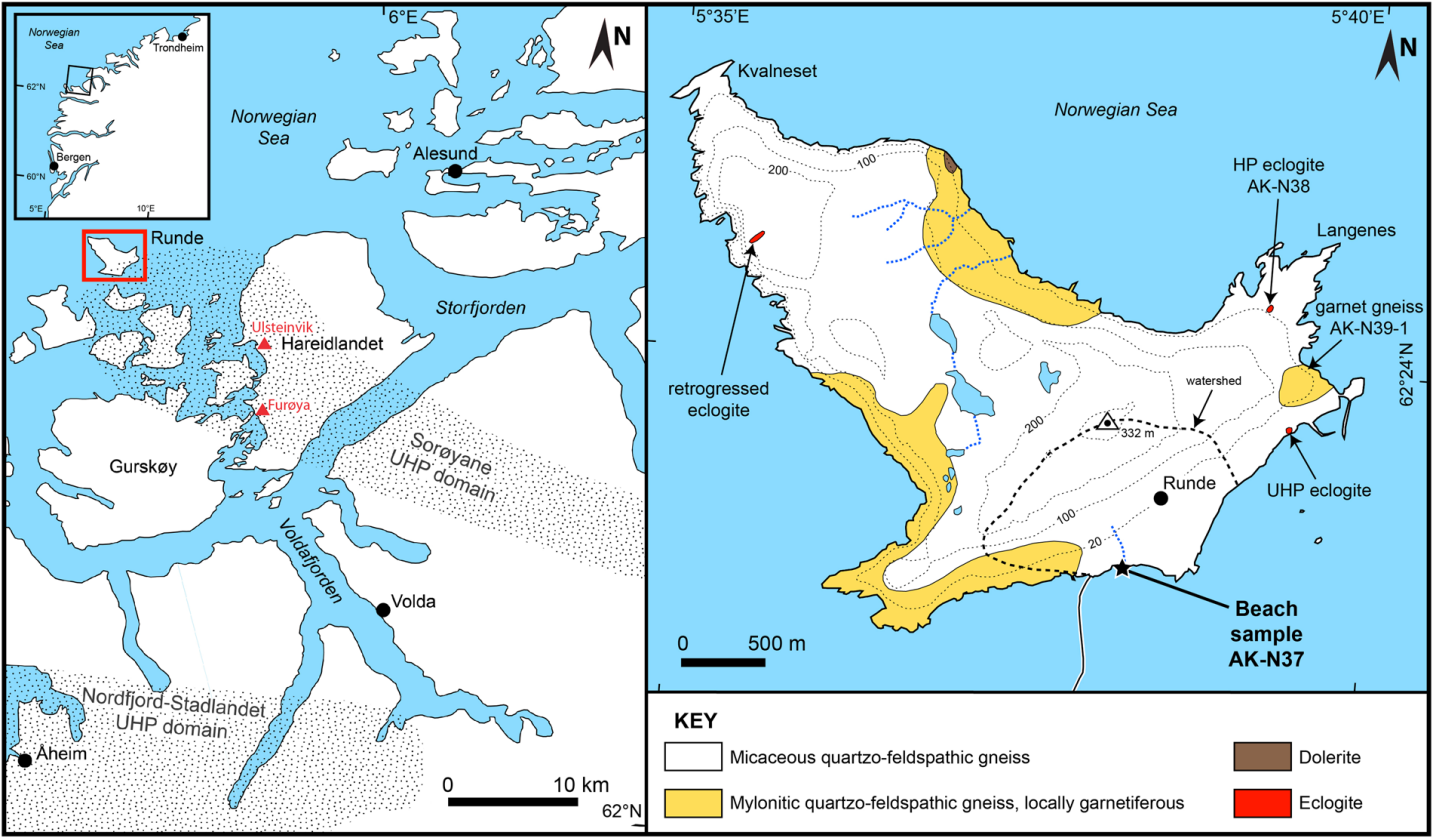
Map showing the location of the studied area and the sampling location (modified from Krippner *et al*.^25^). Left side shows an overview map of the region around Runde (indicated by the red box) including the locations of UHP domains. Triangles mark known UHP occurrences southeast of Runde^26,45^. Right side shows the geology of Runde and the location of the beach-sediment and crystalline rock samples^25^.

Here we present a new approach to detecting UHP metamorphic rocks/terranes. It is based on the first findings of intact monomineralic coesite inclusions in detrital garnet grains from a beach-sediment sample (AK-N37) taken at the mouth of a small stream on the southeast coast of the island of Runde (N62°23.341′, E5°38.252′, Fig. 1)^25^. The island is located at the north-western margin of the Sorøyane UHP domain within the Western Gneiss Region (WGR) of southwest Norway^26^. The new approach avoids numerous drawbacks related to the traditional approach and provides a novel perspective on the prospective exploration of UHP metamorphic rocks/terranes.

## Coesite inclusions in detrital garnet

Overall, 732 detrital garnet grains from three grain-size fractions were analysed for mineral inclusions, 249 from the 63–125 µm fraction, 239 from the 125–250 µm fraction, and 244 from the 250–500 µm fraction. 615 of the 732 analysed garnets (~84%) yield mineral inclusions ≥2 µm, which were identified by Raman spectroscopy with a focus on the presence of UHP mineral inclusions. Six of the analysed garnets exhibit UHP mineral inclusions in the form of 13 intact monomineralic coesite inclusions. Four of the six coesite-bearing garnets (grains number 24, 98, 142, and 209) are from the finest grain-size fraction (63–125 µm), two (grains number 378 and 452) are from the medium fraction (125–250 µm), and no coesite inclusions were detected in the coarse fraction (250–500 µm). Most grains with coesite inclusions are single garnet grains, but grains number 24 and 98 are composed of garnet in contact with plagioclase and quartz, thus represent lithoclasts from their respective source rocks (for simplification in the following also termed as ‘grains’).

All detected coesite inclusions are small (<12 µm) and have a spheroidal or spherical shape. Furthermore, the Raman spectra of all coesite inclusions show a shift of the main band to higher relative wavenumbers compared to the main band position of measured relictic coesite cores in bimineralic SiO_2_ inclusions in ruptured omphacite detected in a thin section from a known UHP eclogite occurrence (AK-N12, N61°58.710′, E5°14.063′)^25^. This UHP eclogite is located at the harbour of Flatraket ~50 km southwest of the studied sample. Experienced UHP conditions are recorded by bimineralic coesite/quartz inclusions (coesite relicts 50–300 µm in size) in garnet^27^ and omphacite^28^ (Supplementary Figure 5), and also by polycrystalline quartz inclusions in garnet and omphacite^27,29^. Both inclusion types show the typical radial expansion fractures originating from the inclusion/host boundary and spreading out into the host mineral (Supplementary Figure 5). The Raman main band shift of the monomineralic coesite inclusion spectra varies between ~1.9 and 3.3 cm^−1^, indicating inclusion overpressures of between ~0.6 and 1.1 GPa (see methods).

**Figure 2:**
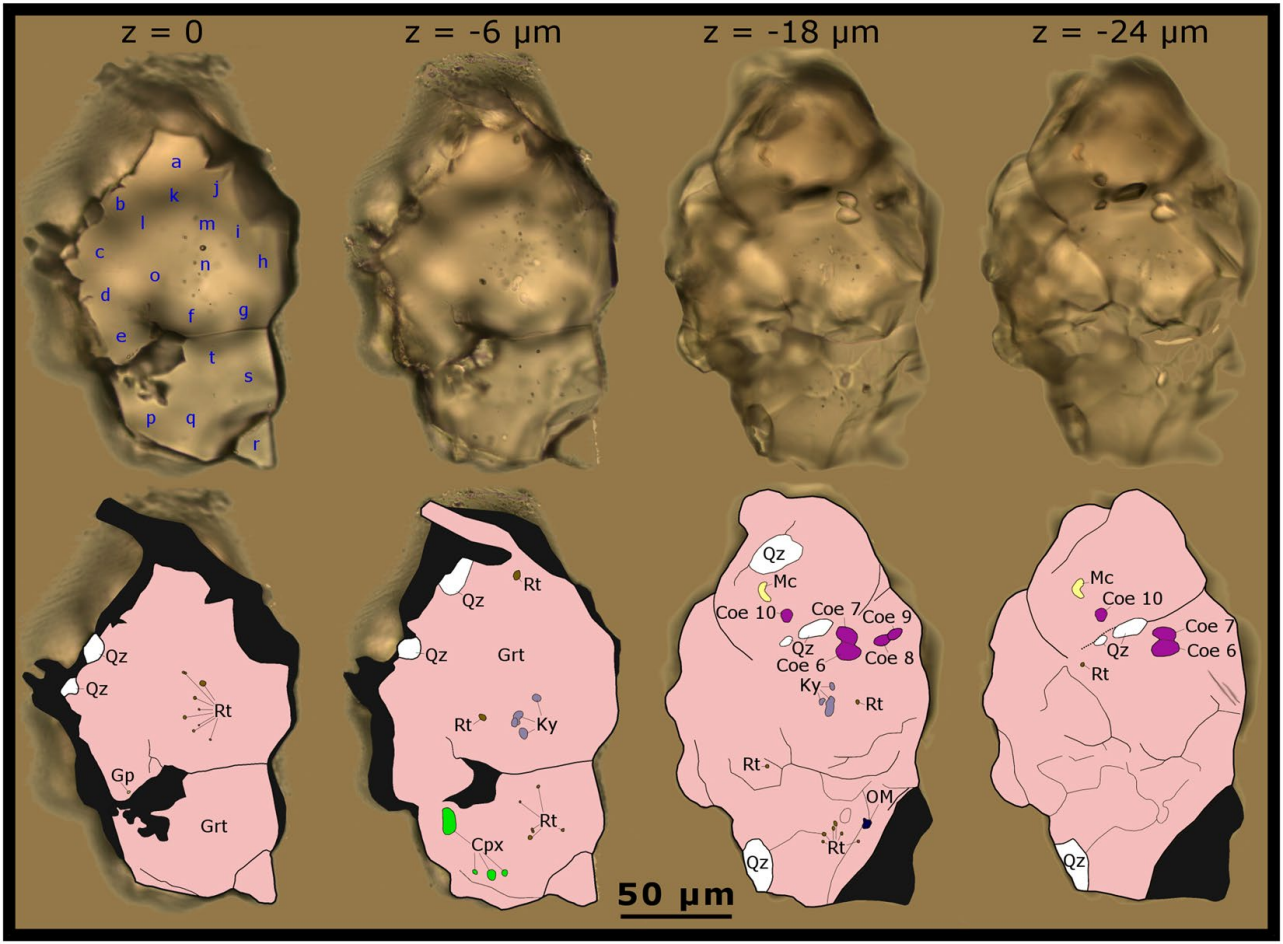
Coesite and other mineral inclusions in detrital garnet (grain number 209). Grain photographed in four different z-positions of the focal plane (z = 0 equates to the polished surface) and corresponding schematic illustrations showing the mineral paragenesis. Out of focus zones are coloured in black. Blue characters indicate electron microprobe measurement spots as designated in Supplementary Table 1. Abbreviations: Coe – coesite; Cpx – clinopyroxene (diopside–omphacite); Grt – garnet; Gp – gypsum; Ky – kyanite; OM – organic matter; Mc – mica (phlogopite–biotite); Qz – quartz; Rt – rutile. Note that techniques which are restricted to the polished surface (z = 0) would miss all detected coesite inclusions.

**Figure 3:**
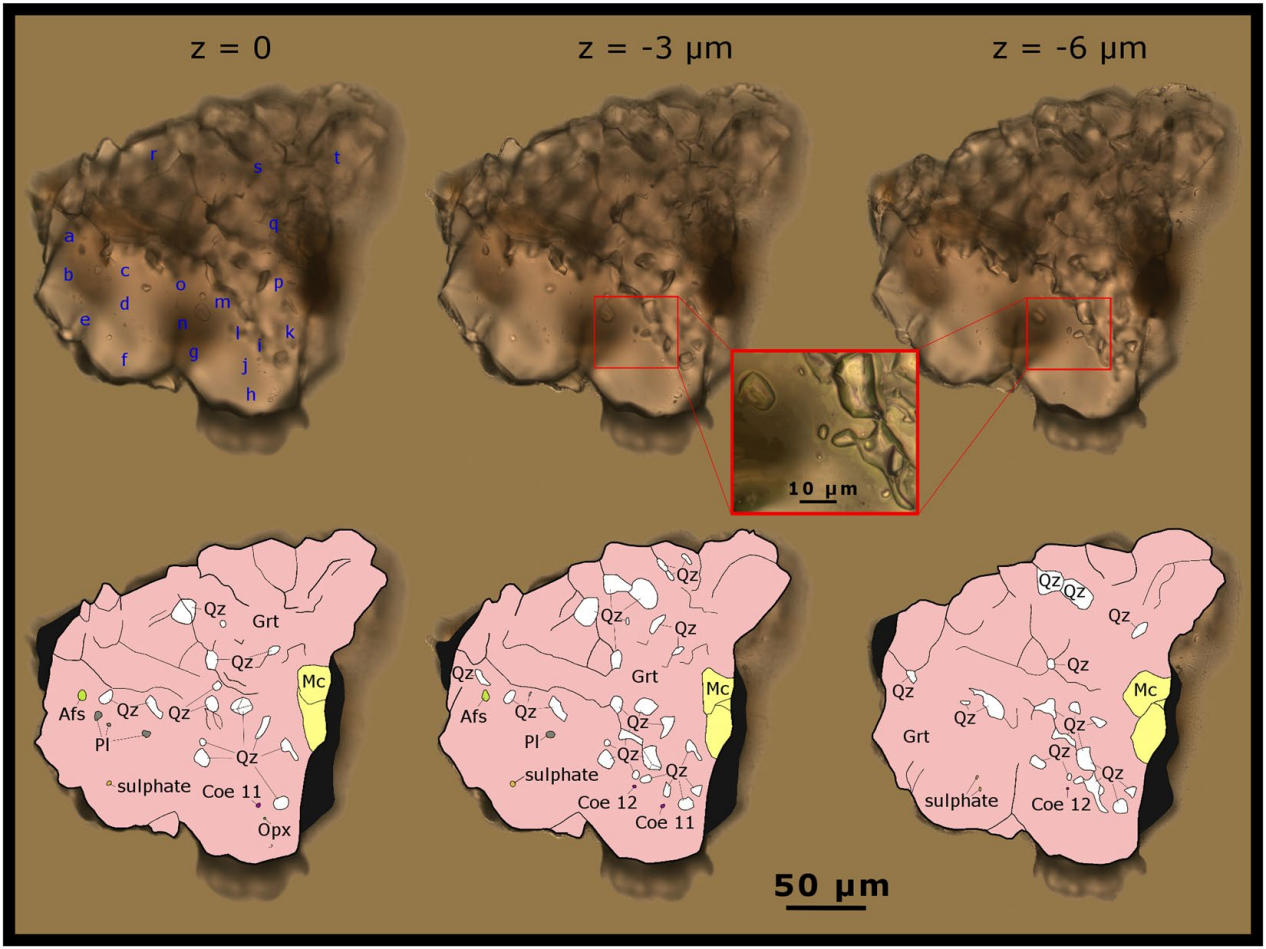
Coesite and other mineral inclusions in detrital garnet (grain number 378). Grain photographed in three different z-positions of the focal plane (z = 0 equates to the polished surface) and corresponding schematic illustrations showing the mineral paragenesis. Out of focus zones are coloured in black. Blue characters indicate electron microprobe measurement spots as designated in Supplementary Table 1. Abbreviations: Afs – alkalifeldspar; Coe – coesite; Opx – orthopyroxene (probably enstatite); Grt –garnet; Mc – mica (phlogopite–biotite); Pl – plagioclase; Qz – quartz.

Except for grain number 24, quartz inclusions are also present in the grains, sometimes very close (<5 µm) to the overpressured intact monomineralic coesite inclusions. All quartz inclusions that are adjacent to coesite are larger than the coesite inclusions, often show fractures originating from the inclusion/host boundary spreading out into the garnet host, and/or are connected to other inclusions by fractures. In Table 1 information about the detected coesite inclusions are listed regarding host grain number, inclusion size, Raman main band position, Raman main band shift, calculated inclusion pressure, and other mineral inclusions within the host grain. Photographs and corresponding schematic illustrations are given for grains number 209 and 378 in different z-positions of the focal plane in Figs 2 and 3. See Supplementary Information for a more detailed description of the coesite-bearing garnets and Supplementary Figs 1–4 for photographs and schematic illustrations of grains number 24, 98, 142, and 452.

**Table 1:** Measured and calculated parameters of coesite inclusions in detrital garnet grains.

Grain number	Coesite number	Coesite size [µm × µm]	Raman main band position [cm^−1^]	Raman shift [cm^−1^]	Calculated inclusion pressure [GPa]	Other mineral inclusions
24	1	5.5 × 2.5	524.0	3.3	1.1	plagioclase
98	2	3.5 × 2.0	523.8	3.1	1.1	quartz, calcite
142	3	3.1 × 2.2	522.9	2.2	0.8	quartz, apatite
	4	5.2 × 2.7	522.9	2.2	0.8	
	5	6.6 × 3.5	523.1	2.4	0.8	
209	6	11.6 × 7.4	523.4	2.7	0.9	quartz, rutile, kyanite, clinopyroxene (diopside–omphacite), mica (phlogopite–biotite), gypsum
	7	9.8 × 6.1	523.2	2.5	0.9	
	8	6.5 × 4.8	523.1	2.4	0.8	
	9	6.4 × 4.1	522.6	1.9	0.6	
	10	6.0 × 5.3	523.1	2.4	0.8	
378	11	2.8 × 1.7	523.1	2.4	0.8	quartz, feldspar, sulphate-mineral, orthopyroxene (probably enstatite)
	12	1.0 × 1.0	523.7	3.0	1.0	
452	13	2.5 × 1.5	523.3	2.6	0.9	quartz

See methods for the procedure to determine the coesite size, the Raman main band position, the Raman shift, and the calculated inclusion pressure.

**Figure 4:**
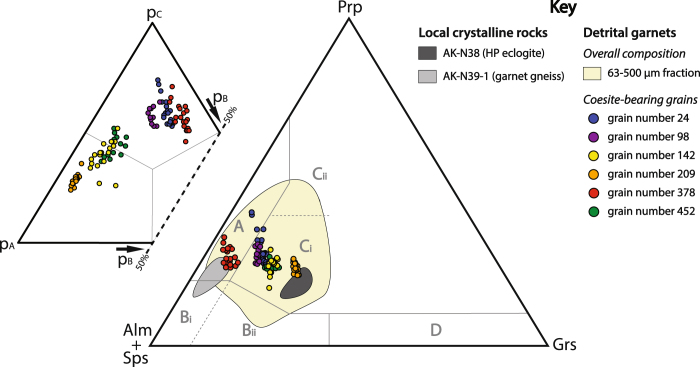
Geochemical garnet compositions of detrital grains and two local crystalline rocks. The right (large) ternary diagram considers the main endmembers almandine (Alm), spessartine (Sps), grossular (Grs), and pyrope (Prp), according to the classification diagram for metamorphic garnets^30^. This includes the discrimination fields for granulite-facies metasediments (A), amphibolite-facies metasediments (Bi + Bii), intermediate acidic igneous rocks (Bi), high-grade metamafic rocks (Ci), ultramafic rocks (Cii), metasomatic rocks and others (D). The distribution of data points for the crystalline rocks AK-N38 (eclogite, 58 data points^25^) and AK-N39–1 (garnet gneiss, 116 data points^25^) are shown by envelopes. The overall detrital garnet composition of the grain-size fraction 63–500 µm (49 data points from 63–125 µm fraction, 47 from the 125–250 µm fraction, and 49 from the 250–500 µm fraction) is outlined by the light yellow field (one outlier excluded). Filled cycles show the composition of the coesite-bearing garnets at several measurement points (see Figs 2 and 3, and Supplementary Figs 1–4). For each of these compositions the probabilities of belonging to the major metamorphic host-rock groups (p_A_ = eclogite-facies rocks, p_B_ = amphibolite-facies rocks, p_C_ = granulite-facies rocks) have been calculated and plotted (small ternary diagram in the upper left) using ‘equal-M’ as prior probability^31^.

Geochemically, the six coesite-bearing garnet grains are within the overall composition of detrital garnets from the beach sample, given by the geochemical variability of ~50 analysed garnet grains from each of the three grain-size fractions (Fig. 4, Supplementary Table 1). Thus, the garnet grains with coesite inclusions cannot be geochemically distinguished from the bulk of detrital garnets. The coesite-bearing garnets, however, show compositional variation among each other, particularly regarding grossular component. Within the single garnet grains, compositional variation is mainly controlled by variation in pyrope content.

In the classical ternary discrimination diagram for metamorphic garnets^30^ (Fig. 4, right side), composition of grain number 209 plots well within the field of high-grade metamafic rocks (Ci), while composition of grain number 378 plots in the field of granulite-facies metasediments (A). The other coesite-bearing grains represent intermediate compositions between grain number 209 and 378, with grain number 142 and 452 closer to grain number 209 (also field Ci), and grain number 24 and 98 closer to grain number 378 (transition zone between fields Ci and A).

Derivation of the detrital coesite-bearing garnets from high-grade metamorphic source rocks (i.e. eclogite- or granulite-facies rocks) becomes even more obvious when considering a recently developed multivariate statistical discrimination scheme, which assigns to each garnet composition the probability of belonging to the three major garnet-bearing metamorphic facies^31^ (Fig. 4, upper left side). All grains are assigned with highest probability to either eclogite- or granulite-facies rocks. Three grains (number 24, 98, and 378) are assigned to granulite-facies rocks, one grain (number 209) to eclogite-facies rocks and two grains (number 142 and 452) scatter around the boundary between eclogite- and granulite-facies rocks.

## Coesite formation and preservation

As shown by several inclusion/host models, coesite inclusions in garnet cannot be produced from originally captured quartz inclusions (particularly under conditions below the coesite stability field), because the different thermoelastic behaviour of garnet and quartz leads to an inclusion underpressure during prograde metamorphism^16,32–35^. Additionally, even if the quartz inclusion reaches the quartz/coesite equilibrium line (external conditions have to be well above), coesite growth is prevented by the volume decrease during transformation^32^. Consequently, if coesite is present as an inclusion in garnet, it must have been present as a matrix phase during garnet growth under *P–T* conditions of the coesite stability field, which is per definition evidence for UHP metamorphism.

Regardless of how precise the determination of the coesite main bands and the translation into inclusion overpressures is, the systematic Raman shift of the coesite main bands to higher relative wavenumbers compared to coesite relicts in inclusions in ruptured hosts is indisputable. Similar characteristics have been observed for intact monomineralic coesite inclusions in garnets of crystalline rocks^22,36,37^. This demonstrates that all detected coesite inclusions are intact and still overpressured, implying that they never ruptured their garnet host and never equilibrated with the external metamorphic conditions after UHP metamorphism.

Notably, the intact monomineralic coesite inclusions are often located directly adjacent to monomineralic quartz inclusions. This configuration could reflect a capturing of the polymorphs during different metamorphic stages^36,38^. However, because the quartz and coesite inclusions are often very close to each other (sometimes <5 µm separation; Figs 2 and 3) and coesite is sometimes also located at several sites around quartz (Fig. 2), multiple rapidly changing conditions would be necessary to capture the different polymorphs during different metamorphic stages. Furthermore, at all metamorphic stages garnet has to grow, and the isolation of the inclusions has to be very effective by a thin garnet cover of <5 μm. Because of the not existing systematic zonation of quartz-to-coesite, such a scenario is considered rather unlikely. Instead, we suggest that adjacent quartz and coesite inclusions were both originally captured as coesite, but only some were preserved and the others transformed to quartz during exhumation. The dominating factors for the preservation of coesite are the *P–T* conditions at entrapment, thermal properties of the host, the course of the exhumation *P–T* path, and the amount of incorporated fluids^16,20,32,33^. These factors, however, should be very similar for adjacent inclusions and probably cannot explain the presence of coesite inclusions directly next to quartz inclusions. Because all detected coesite inclusions are spheroidal or spherical, their longest axes are always <12 µm, and adjacent quartz inclusions are always larger and/or connected to larger grains by fractures, we assume that the small inclusion size and the spheroidal/spherical inclusion shape are responsible for their preservation. In contrast, inclusions which are larger and/or angular have ruptured their host, equilibrated with the external conditions, and transformed to quartz. Reasons for this are that a larger initial fracture length given by the inclusion size (increasing with size) and higher stress concentrations at corners given by the inclusion shape (increasing with the degree of faceting) reduce the inclusion overpressure necessary to rupture the host^39^.

## Source of the coesite-bearing garnet grains

Because the beach-sediment sample was taken directly at the mouth of a small modern stream^25^ and the absence of any morphological features indicating influences of coastal currents, mixing with material transported along shore can be neglected. Consequently, the sediment was almost exclusively derived from the catchment of the stream (see Fig. 1). However, parts of this material may also have originated from reworked sediments or sedimentary rocks located within the catchment. This would scale up the area where the initial source rocks might have been located. In the sampled catchment, the sedimentary material could have been reworked from glacial or older uplifted beach deposits. Older beach deposits are unlikely to make up a significant amount of eroded material supplied to the modern beach because the southeast coast of Runde represents an uplifted coastal terrace where uncovered local bedrock is exposed the first ~20 m above sea level^40^. In contrast, above that terrace, glacial deposits are known in the form of moraines and erratic blocks^40,41^. In general, moraine deposits in Norway mainly belong to the last glaciation, where the region was covered by the Late Weichselian ice sheet^42–44^. Ice streams which reached and passed Runde originated from the southeast roughly following the drainage route constrained by the Voldafjorden^42^ (Fig. 1, left side). This route passes through parts of the Sorøyane UHP domain, where some UHP rocks are known, like the coesite-bearing eclogites at Ulsteinvik and Furøya^26,45^ (Fig. 1, left side).

Because the crystalline bedrocks of the eastern part of Runde are extensively exposed^46^, the moraine deposits are likely thin and/or restricted to the lower slopes. Therefore, significant contribution of garnet from reworked glacial deposits within the small catchment at Runde is considered unlikely. Reworking within the sampled catchment, however, cannot be entirely excluded and some coesite-bearing garnet may derive from other WGR UHP rocks southeast of the island of Runde. This would imply that the source rocks of the coesite-bearing garnets were initially located within a much larger glacial drainage basin than the small catchment at Runde and, consequently, that the approach introduced here can also be applied to much larger catchments.

However, even if the garnets are derived from reworked sediments, these have to be located within the catchment. To verify the location of the initial UHP garnet source rocks, local crystalline rocks and glacial deposits within the catchment should be tested regarding garnet content and geochemistry, to compare the compositions with that of the detrital coesite-bearing garnets.

Geochemical compositions as well as mineral inclusion assemblages of the garnets with monomineralic coesite inclusions call for more than one UHP lithology as source. Important to note is that the information derived from mineral inclusions in detrital garnets is limited to a mineral assemblage of just two phases, the host garnet and one inclusion. This is because a single garnet grain can record multiple metamorphic stages, which are difficult to distinguish in a detrital fragment. Therefore, the occurrence of diagnostic mineral assemblages using more than one inclusion in garnet can only provide some hints, particularly if only a small number of garnet grains are considered. Based on that, two source rock endmembers can be separated. The first is represented by garnet grain number 209, and the second by grain number 378 and likely grains number 24 and 98. Because of the geochemical assignment to high-grade metamafic rocks in the classical discrimination diagram, the compositional similarity to a local eclogite (AK-N38), the high probability of belonging to an eclogite-facies source considering multivariate statistics (Fig. 4), and the inclusions made of clinopyroxene (diopside–omphacite), kyanite, and rutile, we suggest an eclogitic source rock for garnet grain number 209. At Runde, numerous pods of eclogite occur^46^ which – with a few exceptions – have not yet been mapped. In contrast, grain number 378 shows the geochemical signature of granulite-facies metasediments, high statistical probability of belonging to a granulite-facies source (Fig. 4), and mineral inclusions of orthopyroxene (probably enstatite), feldspar, and numerous quartz, which makes a high-grade felsic source rock much more likely than a mafic (eclogitic) source. The same is suggested for grains number 24 and 98, although the assignments are less definite. Source rock discrimination for grains number 142 and 452 is more difficult because the recorded information is less clear. Geochemical contrasts to both source rock endmembers outlined above, however, suggest another high-grade metamorphic source rock type for these garnet grains.

In summary, our findings of intact monomineralic coesite inclusions in detrital garnet grains represent (i) the first evidence of UHP metamorphism inferred from detrital mineral grains and (ii) the first finding of intact monomineralic coesite in the WGR of Norway. Geochemical composition of the coesite-bearing garnet grains and other mineral inclusions besides coesite call for more than one crystalline source lithology, most likely including felsic crystalline rocks. The sources of the coesite-bearing garnets were exposed within the small catchment area at Runde, but may include glacial deposits, which in turn could have been sourced from a larger region of the WGR. Because of the minor abundance of glacial deposits in the catchment area compared to the extensively exposed crystalline rocks, we suppose that most (if not all) of the coesite-bearing garnets are derived from crystalline rocks exposed within the local catchment. This would imply that at least one additional UHP source rock exists on the island of Runde because, to date, only one UHP locality is known from the island of Runde (eclogite pod with bimineralic coesite/quartz inclusions in garnet), which is located east of the catchment area of the sediment sample investigated^25,26^ (Fig. 1).

Regardless of the small size of the study area, we have demonstrated that focusing on detrital minerals provides a complimentary and effective approach to capture the distribution and characteristics of UHP rocks exposed at the surface at the time of sediment generation and deposition. Although, some uncertainties regarding the location of the initial UHP source rocks within the sampled catchment may exist, particularly in regions affected by glacial processes, the applied method is capable of tracing UHP metamorphic rocks and/or their erosional products at the catchment scale.

## Implications for tracing ultrahigh-pressure terranes/rocks

Tracing UHP metamorphic terranes/rocks by analysing the inclusions of detrital minerals such as garnet has major advantages over looking for petrographic evidence in crystalline rocks. First of all, large catchment areas can be covered systematically with a few sediment samples, allowing the detection of UHP metamorphism in large rock volumes that equilibrated only locally under UHP conditions (for example, due to small-scale fluid infiltration^47^). Identifying these locations in crystalline rocks can be very challenging and time-consuming, particularly in large regions and when the rocks are strongly altered, covered by soil, or otherwise poorly accessible. Moreover, UHP metamorphic rocks frequently undergo multi-phase metamorphism, and the earlier stages are typically not visible in the field^37^. Many potential UHP rocks, especially felsic rocks, were likely never analysed regarding UHP metamorphism and even they were, sampling the right location may be just serendipity. In contrast, by analysing the detritus, we get a mixture of garnets from a much larger volume of garnet-bearing rocks in the catchment area, whereby grains from overprinted rocks are included. Earlier UHP stages can be preserved therein because garnet can record several growth zones representing different metamorphic stages or events with accompanied mineral inclusions^36,48–51^. The method furthermore enables the detection of UHP terranes that have once been present at the Earth’s surface but were subsequently eroded and buried, by analysing garnet grains from clastic sedimentary rocks. Garnet is a comparatively stable heavy mineral during transport, surface weathering, and deep burial conditions^52^. The presented approach has significant advantages when seeking for monomineralic coesite inclusions. Sediment samples are probably enriched in garnets containing these inclusions because coesite inclusions which (partially) transformed to quartz will likely have ruptured the garnet host, resulting in a disintegration of the garnet grain during weathering and transport. Furthermore, the approach can also be applied to other heavy minerals such as zircon and rutile, which are both capable of hosting mineral inclusions recording UHP conditions^22,37,53–55^.

These new capabilities are of special interest for tracing hitherto unknown UHP terranes in which the UHP metamorphic stages are obscured by overprinting, where UHP indicators have been overlooked so far due to the absence of typical structures (for example, pseudomorphs after coesite in the sampled crystalline rocks), or which may be recorded in sedimentary rocks only due to complete erosion or burial. Thus, we consider that analysing inclusions in detrital mineral grains concerning UHP mineral indicators have the highest potential for tracing ancient UHP terranes and irrefutably clarifying the question if deep subduction processes were established prior to the Neoproterozoic as suggested by several authors^12–15^.

## Methods

### Mineral separation and sample preparation

Mineral separation and sample preparation were performed at the University of Göttingen (Geosciences Centre, Department of Sedimentology and Environmental Geology). The sediment sample was wet sieved to separate grain-size fractions, treated with acetic acid, split by quartering, and the heavy mineral fraction was separated using sodium polytungstate with a density of 2.89 g cm^–3^. Garnets were handpicked under the binocular microscope from three grain-size fractions (63–125 µm, 125–250 µm, 250–500 µm) and embedded in synthetic mounts using a bonding epoxy composed of a mixture of Araldite® resin and hardener at a ratio of 5:1. Mounts with the picked garnet crystals were ground with silicon carbide abrasive paper and polished in two steps with 3 µm and 1 µm Al_2_O_3_ abrasives in suspension.

### Raman spectroscopy

Raman spectroscopy was performed at the University of Göttingen (Geosciences Centre, Department of Sedimentology and Environmental Geology) using a Horiba Jobin Yvon XploRA Plus spectrometer equipped with an Olympus BX41 microscope, a 532 nm diode laser (20–25 mW maximum output power) and a motorised x-y-z stage. The confocal microscope is coupled to a 200 mm focal length spectrograph equipped with a four-grating turret (2400 l mm^−1^, 1800 l mm^−1^, 1200 l mm^−1^, and 600 l mm^−1^). All measurements were performed using a 100× objective with a numerical aperture of 0.9. The confocal hole diameter and slit were set to 100 µm. For mineral identification of all inclusions ≥2 µm within the garnets, the 1800 l mm^−1^ grating was used, the spectrometer was calibrated on the 520.70 cm^−1^ line of Si, and the recorded spectrum was centred at 1000 cm^−1^. Inclusions <2 µm were sometimes also identified if they had a good Raman response. Captured spectra were exported to the software CrystalSleuth^56^, the background was automatically subtracted via the software, and the corresponding mineral was identified by comparison with the RRUFF database^57^ also via the CrystalSleuth software. Some of the identified minerals were merged into main groups. Overall, the performed analysis of the inclusions needed ~150 hours of work in the Raman laboratory (~5 garnet grains per hour).

To verify the systematic shift of the coesite inclusions main bands, the Raman spectra of all identified coesite inclusions were captured again using a specific calibration and correction method. To achieve the highest resolution, the 2400 l mm^−1^ grating was used, the spectrometer was calibrated on the 520.70 cm^−1^ line of Si, and the centre of the spectrum was left in the same position like during the Si-standard calibration (520.62 cm^−1^). This position is very close to the position of the coesite main band at ~521 cm^−1^ at atmospheric pressure and room temperature^22,58^. Due to possible small inaccuracies of the Si-standard calibration, a possible drift during the measurement session and/or a possible stretching or compression of the spectral field, a correction of the coesite main band position was performed using distinctive spectral lines of a neon glow lamp as reference positions. The light of the neon glow lamp was captured simultaneously (or directly afterwards) to the capturing of the spectrum from every single coesite inclusion. For that, the acquisition time of every measurement was set to 120 seconds and two accumulations. This time interval is high enough to get a sufficient intensity of the coesite main band using a laser power of only ~1 mW. The laser power was reduced sufficiently to reduce the interference of the coesite spectrum and the neon spectrum enabling the simultaneous capturing. Only for three small coesite inclusions (coesite number 3, 12, and 13), it was not possible to capture the coesite spectrum and the neon spectrum simultaneously, because the intensities of the coesite inclusions were too low compared to the neon lines. These three inclusions were measured with ~10 mW laser power, and the neon spectrum was captured directly afterwards. Spectra evaluation was performed within the Labspec 6.4.4 software by cutting the spectra between 373 and 837 cm^−1^, subtracting the dark-noise of the charge-coupled device detector, subtracting the background by a polynomial baseline fit, adding peaks at four selected neon reference line positions and the coesite main band, and fitting the peaks to the captured spectra using a Gaussian Lorentzian mixed function (pseudo-Voigt). Selected neon reference line positions are at 543.365 nm (equal to 386.098 cm^−1^ relative), 544.851 nm (equal to 436.284 cm^−1^ relative), 555.910 nm (equal to 801.398 cm^−1^ relative), and 556.244 nm (equal to 812.212 cm^−1^ relative). The measured main band position of every coesite inclusion was corrected by a linear regression function based on the difference between the measured neon line positions compared to their reference positions. In the same way, the main band positions of two coesite cores in biminineralic coesite/quartz inclusions in ruptured omphacite of the UHP eclogite at Flatraket harbour (sample AK-N12^25^; Supplementary Fig. 5) were determined. Because the omphacite hosts are ruptured, the inclusion pressures are released, and the coesite spectra reflect atmospheric pressure conditions. The difference between the coesite main band positions from the intact monomineralic coesite inclusions in the detrital garnets compared to the main band positions of the coesite cores in the Flatraket UHP eclogite (≤520.7 cm^−1^) reflect the main band shift due to inclusion overpressure. These overpressures were calculated using the experimentally determined ratio of 1 GPa per 2.9 cm^−1^ ^58^. The inclusion size of the coesite inclusions (see Table 1) was determined within the Labspec 6.4.4 software using the 100x objective. Stated are the long and short axes in a two-dimesional (plane) view as situated in the garnet grains and embedded in the epoxy.

### Electron microprobe measurements

Electron microprobe measurements were performed at the University of Göttingen (Geosciences Centre, Department of Geochemistry) using a JEOL JXA 8900 RL electron microprobe equipped with five wavelength dispersive spectrometers. Before analysis, all samples were coated with carbon to ensure conductivity. Measurement conditions include an accelerating voltage of 15 kV and a beam current of 20 nA. Counting times were 15 seconds for Si, Mg, Ca, Fe and Al, and 30 seconds for Ti, Cr and Mn. The compositions of the first ~50 detrital garnet grains from each of the three grain-size fractions (63–125 µm; 125–250 µm; 250–500 µm) and the compositions of the coesite-bearing garnets at several spots were determined. From the measured wt% the molar endmember values were recasted^59^. Multivariate statistics were performed using the prior probability ‘equal-M’^31^.

### Data Availability

All data pertinent to this study and its reported findings can be found in the article itself or the corresponding Supplementary Information file.

## Electronic supplementary material

Supplementary Information
