# Supplementary material for: Tracing ultrahigh-pressure metamorphism at the catchment scale
Source: Sci Rep. 2018 Feb 13;8:2931. doi: 10.1038/s41598-018-21262-8 (PMC5811555; doi:10.1038/s41598-018-21262-8)
Supplement: Supplementary file 1 — Supplementary Information [file 41598_2018_21262_MOESM1_ESM.pdf]

Tracing ultrahigh-pressure metamorphism at the catchment scale

J. Schönig[1], G. Meinhold[1,2], H. von Eynatten[1] & N. K. Lünsdorf[1]

[1]Department of Sedimentology and Environmental Geology, Geoscience Centre Göttingen, University of Göttingen, Goldschmidtstraße 3, 37077 Göttingen, Germany

[2]School of Geography, Geology and the Environment, Keele University, Keele, Staffordshire, ST5 5BG, UK

Correspondence should be addressed to J.S. (e-mail: jan.schoenig@uni-goettingen.de)

Supplementary Information

Description of the coesite-bearing detrital garnets

Garnet grain number 24 (Supplementary Fig. 1) is intergrown by plagioclase and quartz. One spheroidal coesite inclusion of 5.5 µm × 2.5 µm in size is present close to the garnet rim. The coesite main band of the Raman spectrum is located at ~524.0 cm^{-1}, which represents a shift to higher relative wavenumbers compared to the main band position of measured relictic coesite cores in bimineralic SiO_2 inclusions in ruptured omphacite detected in a thin section from a known UHP eclogite. The shift of ~3.3 cm^{-1} is equal to inclusion overpressures of ~1.1 GPa (see methods). Close to the coesite inclusion (<5 µm), one almost idiomorphic plagioclase crystal is present within the garnet. This plagioclase looks like an inclusion, but it is probably connected to the lower surface of the garnet and thus, also represents an intergrown mineral. In addition, two inclusions (probably also not completely enveloped by

garnet) occur at or very close to the lower surface of the garnet, with Raman spectra being typical for organic matter with a very low maturity.

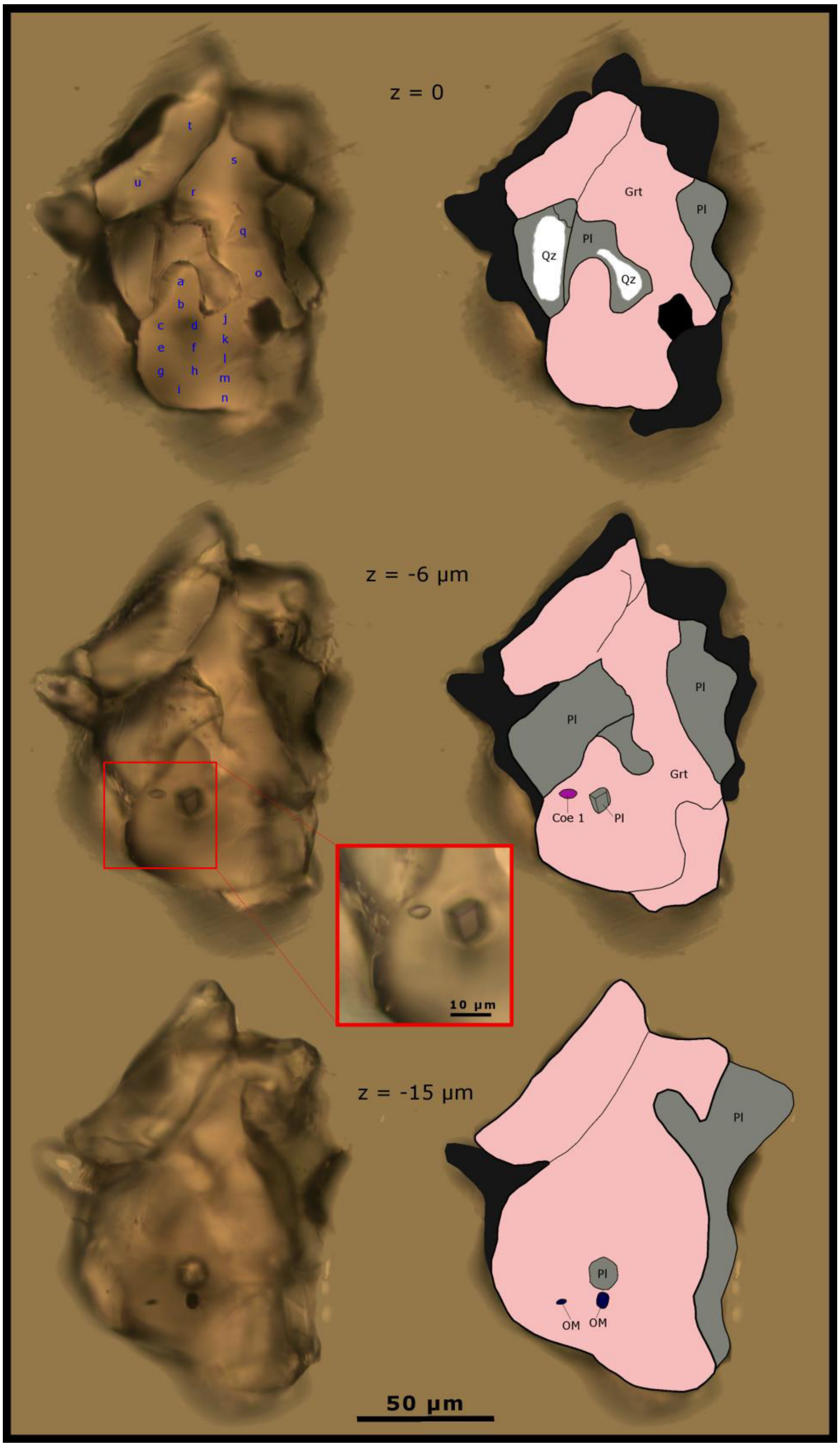

Supplementary Figure 1: Coesite and other mineral inclusions in detrital garnet (grain number 24). *Grain photographed in three different z-positions of the focal plane (z = 0 equates to the polished surface) and corresponding schematic illustrations showing the mineral paragenesis. Out of focus zones are coloured in black. Blue characters indicate electron microprobe measurement spots as designated in Supplementary Table 1. Abbreviations: Coe – coesite; Grt – garnet; OM – organic matter; Qz – quartz; Pl – plagioclase.*

Similar to grain number 24, also grain number 98 (Supplementary Fig. 2) does not consist solely of garnet. Intergrown plagioclase and quartz separate three garnet segments. It is not allocatable if the garnet segments were primarily connected to each other, but because they are connected to a lithoclast, they must originate from the same source lithology. One spheroidal coesite inclusion of 3.5 μm × 2.0 μm in size is present in one of the segments. The coesite main band of its Raman spectrum is located at ~523.8 cm^{-1}, representing a shift to higher wavenumbers by ~3.1 cm^{-1}, which indicates inclusion overpressures of ~1.1 GPa (see methods). In the other garnet segments, several inclusions of quartz occur, which are larger than the coesite inclusion and at least one of the quartz inclusion shows fractures originating from the inclusion/host boundary and spreading out into the garnet host until encountering the segment rims. Besides the quartz inclusions, also a small (~2 μm in diameter) calcite inclusion is present in one of the segments.

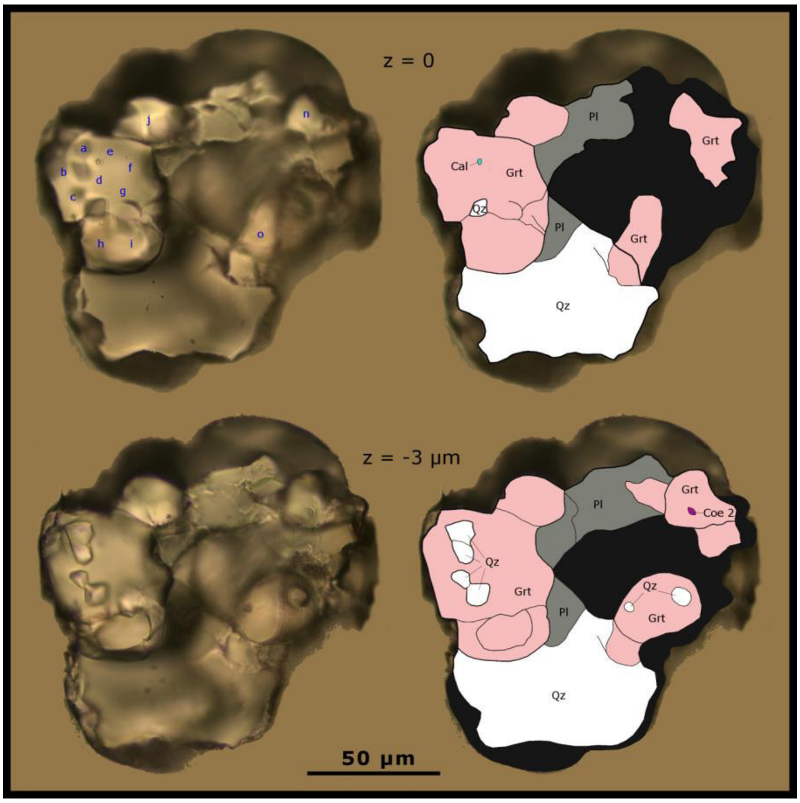

Supplementary Figure 2: Coesite and other mineral inclusions in detrital garnet (grain number 98). *Grain photographed in two different z-positions of the focal plane (z = 0 equates to the polished surface) and corresponding schematic illustrations showing the mineral paragenesis. Out of focus zones are coloured in black. Blue characters indicate electron microprobe measurement spots as designated in Supplementary Table 1. Abbreviations: Cal – calcite; Coe – coesite; Grt – garnet; Qz – quartz; Pl – plagioclase.*

Grain number 142 (Supplementary Fig. 3) represents a single garnet grain, whereby three spheroidal coesite inclusions occur close to its lower surface. The first coesite inclusion is 3.1 µm × 2.2 µm in size and the main band is located at ~522.9 cm^{-1}, the second is 5.2 µm × 2.7 µm in size with the main band at ~522.9 cm^{-1}, and the third is 6.6 µm × 3.5 µm in size with the main band position of ~523.1 cm^{-1}. Thus, the shift to higher relative wavenumbers of between ~2.2 and 2.4 cm^{-1} indicates inclusion overpressures of ~0.8 GPa (see methods). In addition to coesite, also few inclusions of apatite and quartz are present. In the vicinity of the coesite inclusions, the quartz inclusions are larger than the coesite inclusions, but more distant also a single smaller quartz inclusion occurs. Furthermore, two fluid inclusions (CO_2) are present close to the lower surface of the garnet grain.

As grain number 142, also grain number 209 (Fig. 2) represents a single garnet grain, but it contains a different configuration of mineral inclusions occurring together with coesite compared to the grains mentioned before. Overall, five coesite inclusions are present within the garnet, which are located close to each other and have a spherical to spheroidal shape. The first coesite inclusion is 11.6 µm × 7.4 µm in size and the main band is located at ~523.4 cm^{-1}, the second is 9.8 µm × 6.1 µm in size with the main band at ~523.2 cm^{-1}, the third is 6.5 µm × 4.8 µm in size with the main band position of ~523.1 cm^{-1}, the fourth measures 6.4 µm × 4.1 µm with the main band at ~522.6 cm^{-1}, and the fifth is 6.0 µm × 5.3 µm in size with the main band position of ~523.1 cm^{-1}. Thus, the shift to higher relative wavenumbers of between ~1.9 and 2.7 cm^{-1} indicates inclusion overpressures of between ~0.6 and 0.9 GPa (see methods). Additionally, several quartz inclusions are present, whereby two of them are directly surrounded by the coesite inclusions and at the closest point coesite and quartz are isolated from each other by garnet of <5 µm thickness. However, while all of the coesite inclusions are completely intact, all quartz inclusions are either located directly at the rim (not completely enclosed in garnet), or they are connected to the rim by fractures. Besides coesite and quartz, numerous small inclusions of rutile, several inclusions of kyanite, few inclusions

of clinopyroxene (diopside–omphacite), a single mica (phlogopite–biotite) inclusion, and a single gypsum inclusion occur. In addition, one inclusion of organic matter with a very low maturity is located directly at the lower surface, which is probably not completely enveloped by garnet, similar to the organic matter inclusions in grain number 24.

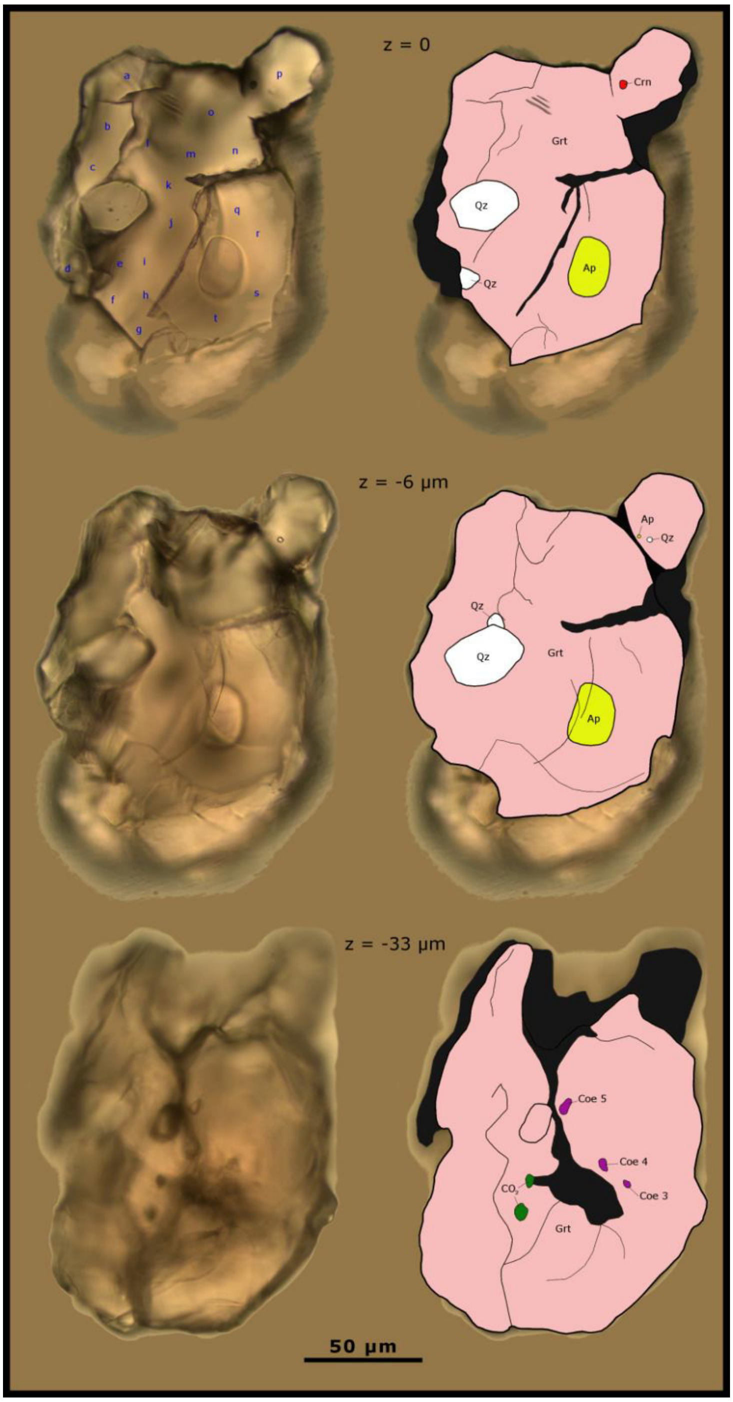

Supplementary Figure 3: Coesite and other mineral inclusions in detrital garnet (grain number 142). *Grain photographed in three different z-positions of the focal plane (z = 0 equates to the polished surface) and corresponding schematic illustrations showing the mineral paragenesis. Out of focus zones are coloured in black. Blue characters indicate electron microprobe measurement spots as designated in Supplementary Table 1. Abbreviations: Ap – apatite; Coe – coesite; Crn – corundum; Grt – garnet; Qz – quartz.*

Grain number 378 (Fig. 3) also represents a single garnet grain. Within that garnet two coesite inclusions occur. The first coesite inclusion has a spheroidal shape, is 2.8 µm × 1.7 µm in size, and the coesite main band is located at ~523.1 cm^{-1}, indicating a shift to higher relative wavenumbers of ~2.4 cm^{-1} equal to an inclusion overpressure of ~0.8 GPa. The second coesite inclusion has a spherical shape, a diameter of 1.0 µm, and its main band is located at ~523.7 cm^{-1}, indicating a shift of ~3.0 cm^{-1} equal to an overpressure of ~1.0 GPa (see methods). Besides coesite, numerous inclusions of quartz, few inclusions of feldspar, few inclusions of a sulphate-mineral, and a single very small orthopyroxe (probably enstatite) inclusion occur. The quartz inclusions are larger than the two coesite inclusions, and often they are also cross-linked with each other. Several of the quartz inclusions show fractures originating from the inclusion/host boundary. In addition to the inclusion types, several mica (phlogopite–biotite) fragments are present at the garnet rim and lower surface, probably representing a contact phase from the source rock.

Grain number 452 (Supplementary Fig. 4) is also a single garnet grain which contains one spheroidal coesite inclusion. The coesite inclusion is 2.5 µm × 1.5 µm in size, and the Raman main band is located at ~523.3 cm^{-1}, indicating a shift to higher relative wavenumbers of ~2.6 cm^{-1} equal to an inclusion overpressure of ~0.9 GPa (see methods). Besides coesite, several quartz inclusions are present, which are all larger than the coesite inclusion and mainly located at the garnet rim.

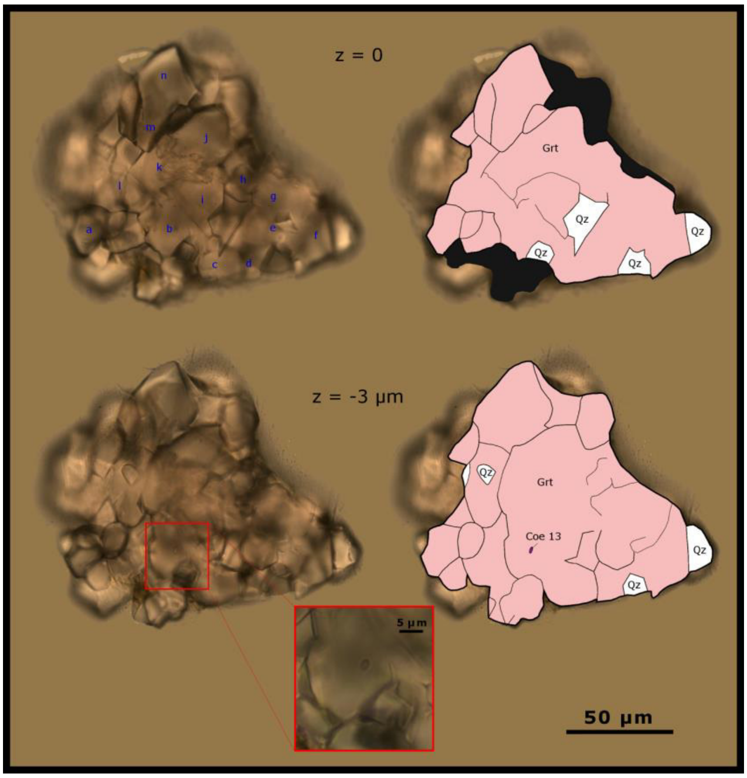

Supplementary Figure 4: Coesite and other mineral inclusions in detrital garnet (grain number 452). *Grain photographed in two different z-positions of the focal plane (z = 0 equates to the polished surface) and corresponding schematic illustrations showing the mineral paragenesis. Out of focus zones are coloured in black. Blue characters indicate electron microprobe measurement spots as designated in Supplementary Table 1. Abbreviations: Coe – coesite; Grt – garnet; Qz – quartz.*

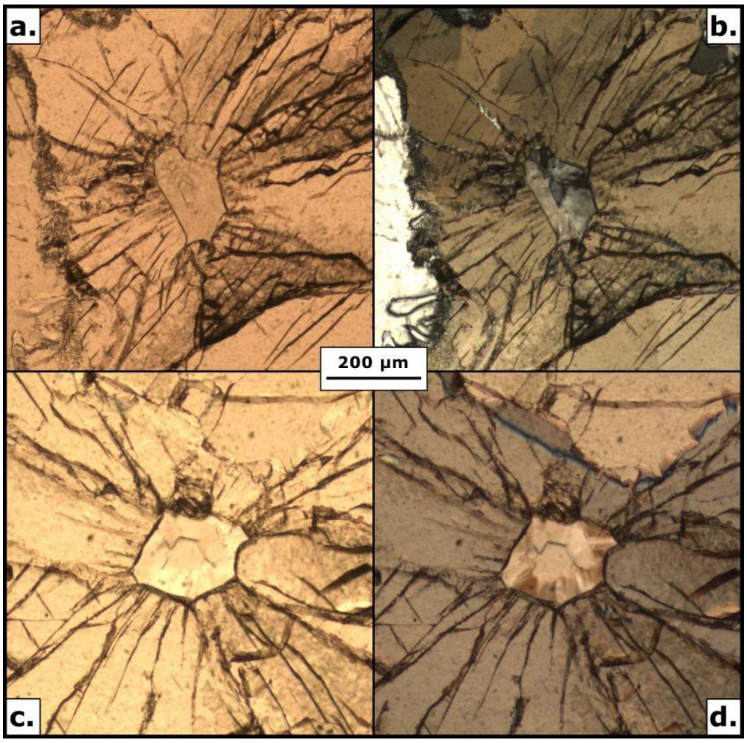

Supplementary Figure 5: Bimineralic coesite/quartz inclusions in ruptured omphacite of the UHP eclogite at Flatraket harbour (sample AK-N12[25]). *The two inclusions are shown in transmitted light (a and c) and with crossed polarizers (b and d). Both show a relictic coesite core and a fine-grained palisade quartz rim. Note the bimineralic inclusions are significantly larger than the intact monomineralic inclusions detected in the detrital garnets of Runde.*

Supplementary Table 1: Geochemical garnet compositions. *Electron microprobe data and calculated main endmembers [59]. Grain number 1–209 from the 63–125 µm grain-size fraction, number 352–452 from the 125–250 µm fraction, and number 706–755 from the 250–500 µm fraction. Abbreviations: Alm – almandine; Sps – spessartine; Prp – pyrope; Grs – grossular.*

Grain number	electron microprobe data [wt%]								normalised endmembers calculated[59] [mol%]			
	SiO_2	MgO	CaO	TiO_2	FeO_{total}	Al_2O_3	Cr_2O_3	MnO	Alm	Sps	Prp	Grs
1	38.640	6.580	9.980	<0.023	22.300	21.880	<0.027	0.421	46.570	0.939	25.694	26.797
2	39.690	10.370	9.210	0.030	16.880	22.240	0.268	0.539	35.524	1.166	39.428	23.882
3	38.320	8.160	3.180	<0.023	26.740	21.800	<0.026	1.660	56.338	3.714	32.113	7.836
4	37.940	4.700	9.550	0.050	24.620	21.220	<0.026	1.420	52.753	3.243	18.913	25.090
5	37.050	1.526	9.860	0.044	27.360	21.100	<0.027	2.590	60.082	5.980	6.194	27.745
6	37.270	7.110	3.240	<0.023	28.150	21.600	<0.026	1.350	59.605	3.085	28.640	8.669
7	38.000	8.660	2.120	<0.023	27.600	21.610	<0.027	1.010	58.572	2.278	34.431	4.719
8	38.740	7.190	10.440	0.052	21.120	21.570	<0.026	0.287	44.072	0.641	28.403	26.884
9	38.600	6.190	10.610	<0.023	22.620	21.610	0.048	0.543	46.711	1.226	24.566	27.497
10	38.390	7.170	6.460	<0.024	25.150	21.780	<0.026	0.949	52.752	2.122	28.232	16.894
11	37.350	3.080	7.450	0.044	29.690	21.180	<0.026	1.430	64.337	3.303	12.521	19.839
12	38.530	7.880	4.040	0.043	27.070	21.660	<0.026	0.958	57.340	2.148	31.075	9.437
13	37.980	6.710	4.390	<0.023	26.980	21.690	0.035	1.770	57.686	3.975	26.495	11.844
14	37.880	4.960	7.470	<0.023	27.200	21.450	<0.027	0.790	58.353	1.790	19.785	20.072
15	38.250	7.590	4.710	<0.023	27.390	21.140	<0.027	0.789	57.732	1.820	30.791	9.657
16	39.440	11.270	7.700	<0.022	18.380	22.110	0.034	0.421	36.488	0.933	43.692	18.887
17	38.130	5.710	7.790	<0.023	26.170	21.580	<0.027	0.597	55.355	1.346	22.693	20.606
18	38.840	6.730	6.650	0.023	24.630	22.030	<0.027	1.790	52.222	3.918	25.954	17.905
19	37.970	6.900	4.770	<0.023	27.530	21.500	<0.028	1.062	58.206	2.418	27.625	11.751
20	38.570	6.570	7.930	<0.022	24.760	21.610	<0.026	0.521	52.404	1.169	25.874	20.554
21	37.840	6.630	2.790	<0.023	28.040	21.890	<0.027	2.510	60.360	5.615	26.123	7.902
22	36.720	1.770	8.650	0.031	25.340	20.940	<0.027	5.740	55.184	13.436	7.289	24.091
23	38.400	7.980	4.400	<0.023	26.090	21.850	<0.027	1.201	54.729	2.682	31.341	11.249
24a	38.280	6.320	6.270	<0.023	24.540	21.520	<0.026	2.980	52.137	6.713	25.038	16.112
24b	38.160	6.620	6.020	<0.023	24.420	21.490	<0.027	2.940	51.671	6.644	26.350	15.335
24c	38.620	6.640	5.890	<0.023	24.600	21.880	<0.027	2.780	52.277	6.134	25.800	15.789
24d	37.700	6.820	5.980	<0.024	24.490	21.400	<0.026	2.890	50.799	6.654	27.651	14.896
24e	38.910	6.610	6.200	0.031	24.440	21.950	<0.027	2.680	52.159	5.872	25.484	16.485
24f	37.790	6.990	5.960	<0.023	24.570	21.530	<0.027	2.780	50.513	6.381	28.253	14.854
24g	39.190	6.400	6.390	0.026	24.140	21.770	<0.027	2.860	52.454	6.295	24.431	16.820

Grain number	electron microprobe data [wt%]								normalised endmembers calculated[59] [mol%]			
	SiO_2	MgO	CaO	TiO_2	FeO_{total}	Al_2O_3	Cr_2O_3	MnO	Alm	Sps	Prp	Grs
24h	37.910	6.900	6.210	0.029	24.020	21.360	<0.026	2.760	50.436	6.318	27.816	15.430
24i	38.710	6.330	6.520	0.029	24.340	21.710	<0.027	2.910	51.957	6.457	24.708	16.878
24j	36.020	7.190	5.790	0.026	24.300	20.420	<0.027	2.990	48.858	7.459	31.579	12.104
24k	33.010	7.660	5.630	<0.024	24.480	18.400	<0.027	2.820	46.045	8.347	39.928	5.681
24l	32.580	7.630	5.710	<0.023	24.650	18.260	<0.026	2.860	45.361	8.681	40.735	5.224
24m	34.870	7.490	5.860	<0.023	24.650	19.530	<0.027	2.900	47.550	7.806	35.466	9.177
24n	35.850	7.260	5.860	0.025	24.320	20.370	<0.027	2.980	48.302	7.505	32.190	12.003
24o	37.610	6.890	5.800	<0.023	24.440	21.150	<0.027	3.050	50.930	7.096	28.214	13.760
24q	36.680	6.610	6.330	<0.024	24.300	20.620	<0.027	2.930	50.199	7.093	28.167	14.541
24r	38.500	6.500	6.600	<0.023	23.930	21.570	<0.026	2.690	51.267	6.003	25.527	17.204
24s	37.690	6.780	6.290	<0.023	24.410	21.050	<0.027	3.020	50.519	7.070	27.920	14.490
24t	34.370	7.420	6.150	<0.024	23.890	19.460	<0.027	2.980	45.587	8.110	35.528	10.775
24u	36.860	7.500	5.500	<0.023	24.740	20.760	<0.027	3.080	49.381	7.499	32.146	10.975
25	38.340	6.360	6.650	<0.023	25.060	21.830	<0.027	1.610	53.370	3.572	24.843	18.215
26	37.770	4.980	5.730	0.044	29.630	21.020	<0.027	1.020	63.938	2.360	20.307	13.395
27	38.120	6.650	1.750	<0.023	31.370	21.520	<0.027	1.019	67.659	2.295	26.334	3.712
28	38.740	6.340	9.640	<0.023	22.800	21.860	<0.027	0.657	48.037	1.454	24.675	25.834
29	40.120	13.720	3.770	<0.023	19.390	22.820	<0.025	0.496	38.316	1.063	51.528	9.094
30	38.020	4.930	7.280	0.054	27.750	21.550	<0.027	0.667	59.527	1.504	19.577	19.392
31	38.570	6.380	6.960	<0.023	25.850	21.930	<0.027	0.878	54.504	1.942	24.858	18.697
32	38.470	5.020	9.660	0.032	24.810	21.800	<0.027	0.767	52.562	1.701	19.660	26.077
33	38.440	5.490	7.490	<0.023	26.810	21.800	<0.026	0.585	57.020	1.300	21.475	20.205
34	38.250	5.420	6.690	0.038	26.960	21.730	<0.026	1.320	57.717	2.945	21.284	18.054
35	38.830	6.230	9.930	0.041	21.740	22.050	<0.026	1.870	45.159	4.122	24.171	26.548
36	38.650	6.490	9.740	0.029	22.890	22.180	<0.026	0.583	47.088	1.288	25.209	26.415
37	38.460	7.910	2.050	<0.024	29.120	21.870	<0.027	1.185	61.615	2.631	30.961	4.793
38	39.030	8.320	6.570	0.056	24.340	21.930	<0.027	0.539	50.248	1.199	32.510	16.043
39	38.430	8.130	2.240	<0.024	27.430	22.120	<0.027	1.960	57.897	4.317	31.537	6.249
41	38.320	8.130	2.220	<0.024	27.650	21.730	0.036	1.740	58.926	3.882	31.954	5.237
42	38.610	6.100	8.080	0.083	25.640	21.900	<0.027	0.591	53.693	1.315	23.914	21.079
43	38.810	6.630	7.070	<0.023	25.820	21.910	0.031	0.454	54.606	0.996	25.742	18.656
44	37.970	7.310	5.100	<0.023	25.980	21.480	<0.027	1.580	54.567	3.598	29.331	12.504
45	38.620	8.120	1.630	<0.023	27.640	21.940	<0.027	2.320	59.149	5.100	31.405	4.346

Grain number	electron microprobe data [wt%]								normalised endmembers calculated[59] [mol%]			
	SiO_2	MgO	CaO	TiO_2	FeO_{total}	Al_2O_3	Cr_2O_3	MnO	Alm	Sps	Prp	Grs
46	39.140	9.420	3.760	0.048	25.320	22.170	<0.026	0.622	53.037	1.361	36.197	9.405
47	38.300	6.570	10.170	0.026	22.500	21.710	<0.027	0.771	45.521	1.748	26.266	26.465
48	38.640	6.900	6.860	0.025	24.620	22.000	<0.026	1.570	51.449	3.480	26.878	18.194
49	38.190	8.120	1.194	<0.023	30.180	21.940	<0.027	0.589	63.904	1.312	31.825	2.960
50	37.170	1.472	8.440	0.085	30.370	21.260	<0.028	1.890	66.305	4.354	5.963	23.379
98a	38.220	6.440	5.470	0.026	26.100	21.610	<0.027	1.690	56.296	3.779	25.317	14.608
98b	39.220	6.680	5.170	<0.023	26.680	21.990	<0.026	1.600	57.400	3.481	25.083	14.036
98c	39.240	6.470	5.270	<0.023	26.480	22.110	<0.027	1.570	57.121	3.432	24.882	14.565
98d	38.820	6.910	5.450	<0.024	26.280	21.750	<0.026	1.560	55.853	3.454	26.938	13.754
98e	38.210	6.800	5.220	0.026	26.110	21.520	<0.026	1.670	55.990	3.759	26.912	13.339
98f	37.580	7.390	5.020	<0.023	26.210	21.210	<0.027	1.680	54.417	3.920	30.336	11.327
98g	38.580	7.040	5.340	<0.023	25.830	21.590	<0.027	1.660	55.207	3.702	27.649	13.442
98h	39.060	6.690	5.520	0.029	26.300	22.040	<0.027	1.620	56.127	3.531	25.654	14.688
98i	37.850	7.240	5.160	0.032	25.990	21.520	<0.027	1.640	54.406	3.751	29.125	12.718
98j	37.460	7.490	5.160	<0.022	25.580	21.140	<0.026	1.910	52.976	4.480	30.894	11.650
98n	38.600	7.130	5.080	<0.023	25.140	21.550	<0.027	2.210	54.243	4.917	27.912	12.927
98o	37.580	7.430	5.140	<0.023	25.550	21.390	<0.026	1.690	53.287	3.903	30.171	12.638
142a	38.740	6.680	6.930	0.046	25.160	21.870	<0.026	0.935	53.637	2.067	25.927	18.369
142b	39.200	6.660	6.800	0.051	25.660	22.050	<0.026	0.837	54.709	1.823	25.496	17.971
142c	39.440	6.580	6.770	<0.023	25.510	22.360	<0.026	0.816	54.578	1.771	25.096	18.555
142d	40.160	6.300	7.150	<0.023	24.950	22.780	<0.026	0.791	54.069	1.734	24.341	19.856
142e	42.300	4.180	7.050	<0.023	25.380	23.740	<0.026	0.661	59.670	1.573	17.520	21.236
142f	39.240	6.490	6.820	0.029	25.790	22.210	<0.026	0.564	55.264	1.225	24.792	18.719
142g	39.140	6.610	6.780	<0.023	25.820	22.130	<0.026	0.661	54.951	1.435	25.233	18.381
142h	38.690	6.530	6.740	<0.023	26.210	21.750	<0.027	0.549	55.713	1.222	25.499	17.566
142i	38.670	6.510	6.880	0.049	26.360	22.060	<0.026	0.550	55.208	1.214	25.309	18.270
142j	38.730	6.420	6.830	0.038	26.530	21.770	<0.026	0.515	56.226	1.145	25.107	17.522
142k	38.910	6.270	6.650	0.028	26.430	21.880	<0.027	0.613	56.759	1.340	24.184	17.718
142l	39.730	5.670	6.740	0.041	26.000	22.350	<0.026	0.572	57.368	1.276	22.299	19.057
142m	41.500	5.280	6.930	<0.023	26.100	23.640	<0.027	0.609	57.990	1.375	20.915	19.721
142n	38.370	6.450	7.380	0.024	25.410	21.420	<0.026	0.719	54.051	1.629	25.655	18.666
142o	38.810	6.440	7.100	<0.023	25.770	21.830	<0.026	0.662	54.814	1.461	25.008	18.718
142p	37.990	7.240	6.430	0.040	25.300	21.390	<0.026	0.939	52.909	2.151	29.222	15.718

Grain number	electron microprobe data [wt%]								normalised endmembers calculated[59] [mol%]			
	SiO_2	MgO	CaO	TiO_2	FeO_{total}	Al_2O_3	Cr_2O_3	MnO	Alm	Sps	Prp	Grs
142q	38.330	6.400	7.100	<0.023	25.940	21.570	<0.027	0.658	54.845	1.481	25.375	18.299
142r	37.980	6.560	7.360	0.032	25.150	21.520	<0.026	0.875	52.706	1.990	26.301	19.003
142s	37.970	6.670	7.170	<0.023	25.130	21.540	<0.026	0.954	52.508	2.176	26.725	18.591
142t	38.520	6.560	6.900	0.037	25.840	21.640	<0.027	0.824	54.704	1.849	25.873	17.574
209a	38.030	6.010	9.640	<0.023	23.860	21.350	<0.026	0.549	49.514	1.265	24.276	24.945
209b	38.730	5.860	9.530	0.029	24.070	21.730	<0.027	0.542	50.819	1.208	22.948	25.026
209c	39.240	5.830	9.270	<0.023	24.150	22.030	<0.027	0.558	51.726	1.217	21.763	25.294
209d	39.660	5.650	9.890	0.031	23.710	22.170	<0.026	0.564	50.594	1.217	21.489	26.700
209e	39.120	5.830	9.540	0.026	23.850	22.280	<0.026	0.549	50.532	1.185	22.223	26.059
209f	38.760	6.180	8.930	0.026	23.900	22.200	<0.027	0.506	50.499	1.109	23.773	24.620
209g	37.930	6.200	9.110	0.028	23.990	21.610	<0.026	0.492	49.793	1.120	24.834	24.253
209h	37.560	6.230	8.840	<0.023	24.150	21.360	<0.027	0.522	50.089	1.202	25.342	23.367
209i	37.230	6.310	8.950	0.026	24.240	21.120	<0.026	0.518	49.628	1.224	26.208	22.941
209j	37.720	6.240	9.110	<0.023	24.010	21.360	<0.026	0.523	49.633	1.204	25.356	23.806
209k	38.100	5.960	9.400	<0.023	23.900	21.470	<0.026	0.516	50.165	1.168	23.827	24.840
209l	38.590	5.920	9.660	0.050	23.730	21.750	<0.026	0.520	50.046	1.157	23.231	25.566
209m	38.520	6.130	9.050	0.058	24.070	21.870	<0.026	0.503	50.674	1.113	23.989	24.224
209n	38.610	6.170	8.980	0.030	23.960	21.780	<0.026	0.481	50.855	1.069	24.099	23.977
209o	38.840	5.930	9.480	<0.023	23.720	21.740	<0.027	0.512	50.619	1.126	23.057	25.198
209p	39.020	5.680	9.650	<0.022	23.740	21.910	<0.026	0.508	51.082	1.107	21.395	26.416
209q	38.940	5.990	9.160	<0.023	23.900	21.920	<0.026	0.516	51.063	1.128	23.078	24.731
209r	37.390	6.330	9.240	0.037	23.940	20.850	<0.026	0.532	49.406	1.262	26.447	22.884
209s	38.160	6.280	9.060	0.031	24.060	21.690	<0.027	0.508	49.902	1.149	25.003	23.947
209t	38.650	6.040	9.190	<0.023	23.890	21.890	<0.026	0.544	50.467	1.198	23.503	24.832
352	40.360	13.760	3.650	<0.022	19.710	22.790	<0.025	0.399	39.177	0.848	51.517	8.458
353	40.210	4.730	8.930	0.037	25.100	22.580	0.035	0.555	55.184	1.231	18.543	25.041
354	38.560	7.960	1.177	<0.022	29.590	21.890	<0.026	1.129	63.581	2.483	30.830	3.106
355	38.750	7.670	4.390	0.076	27.900	21.760	<0.027	0.485	58.722	1.084	30.161	10.033
356	38.010	8.510	6.280	0.035	24.390	21.640	<0.026	0.469	49.373	1.075	34.373	15.179
357	37.470	5.850	1.930	<0.023	31.080	21.530	<0.027	1.820	67.293	4.123	23.345	5.240
358	38.850	6.480	6.340	0.029	25.190	22.030	<0.026	1.620	54.191	3.535	24.867	17.406
359	38.350	4.410	7.550	0.043	28.040	21.460	<0.027	0.658	61.116	1.469	17.350	20.065
360	39.110	8.320	7.950	0.047	22.200	22.360	<0.026	0.501	45.758	1.087	31.887	21.268

Grain number	electron microprobe data [wt%]								normalised endmembers calculated[59] [mol%]			
	SiO_2	MgO	CaO	TiO_2	FeO_{total}	Al_2O_3	Cr_2O_3	MnO	Alm	Sps	Prp	Grs
361	38.870	8.470	8.430	<0.023	21.460	22.230	0.082	0.425	43.783	0.932	32.814	22.470
362	39.310	6.840	10.660	0.049	21.420	22.070	<0.026	0.273	45.039	0.599	26.248	28.115
363	38.050	5.780	3.400	<0.023	29.320	21.760	<0.027	2.520	62.543	5.646	22.777	9.034
364	38.030	6.150	2.070	<0.023	29.500	21.880	0.059	2.780	63.989	6.204	24.149	5.658
365	38.780	6.110	9.650	0.028	23.160	21.930	<0.026	0.380	49.354	0.838	23.617	26.191
366	38.720	7.310	7.380	<0.023	25.130	21.380	<0.026	0.468	52.330	1.057	29.222	17.391
367	38.500	6.960	2.570	<0.023	29.790	21.830	<0.027	1.095	63.830	2.425	27.094	6.650
368	38.420	5.830	2.180	<0.023	30.840	21.950	<0.027	2.150	66.556	4.742	22.624	6.078
369	38.440	7.430	2.330	0.027	29.360	21.810	<0.026	1.202	62.637	2.674	29.067	5.622
370	39.340	8.510	7.770	0.037	22.620	22.320	<0.026	0.459	46.245	1.003	32.658	20.094
371	39.550	10.970	5.200	0.027	21.400	22.120	0.256	0.669	44.159	1.463	42.285	12.093
372	39.370	6.660	6.890	<0.023	25.140	22.460	<0.027	0.833	53.859	1.803	25.428	18.910
373	38.630	6.710	3.570	<0.023	28.270	22.010	<0.027	1.700	60.472	3.727	25.897	9.905
374	38.760	7.810	3.100	<0.023	27.650	22.120	<0.027	0.995	59.215	2.178	30.034	8.573
375	38.640	8.040	1.650	<0.023	28.940	21.970	<0.027	0.871	62.492	1.913	31.016	4.578
376	38.680	9.090	4.490	0.035	25.530	22.170	<0.027	0.475	52.237	1.052	35.495	11.216
377	39.360	8.570	6.260	0.050	23.640	22.390	<0.026	0.427	49.709	0.924	32.476	16.891
378a	38.980	6.070	2.980	<0.023	29.210	22.040	<0.027	1.690	64.121	3.753	23.747	8.379
378b	38.910	6.250	3.370	<0.023	28.730	22.280	<0.027	1.720	62.548	3.792	24.257	9.403
378c	38.240	6.500	2.560	<0.023	29.800	21.760	<0.027	1.720	63.979	3.825	25.458	6.739
378d	38.810	6.770	2.520	0.023	29.200	22.010	<0.027	1.750	63.110	3.830	26.080	6.979
378e	38.980	6.590	2.580	<0.023	28.880	22.240	<0.027	1.680	63.288	3.727	25.744	7.241
378f	39.100	6.580	3.470	<0.024	28.500	22.120	0.039	1.700	61.504	3.714	25.312	9.471
378g	39.090	6.850	2.560	<0.023	29.070	22.310	0.037	1.650	62.951	3.618	26.438	6.993
378h	38.450	6.940	2.850	<0.023	28.540	21.840	<0.027	1.610	61.609	3.550	26.908	7.933
378i	38.460	7.620	2.450	<0.023	28.800	21.640	<0.027	1.680	61.063	3.771	30.089	5.077
378j	38.490	7.490	2.640	<0.023	28.640	21.690	0.033	1.650	60.943	3.684	29.459	5.914
378k	37.970	8.180	1.760	<0.023	28.840	21.330	<0.027	1.670	60.940	3.833	33.065	2.162
378l	38.600	7.330	2.280	<0.024	29.390	21.810	<0.027	1.740	62.335	3.869	28.672	5.124
378m	38.490	7.490	1.740	<0.023	29.390	21.920	<0.027	1.620	62.770	3.574	29.093	4.563
378n	38.650	6.520	2.260	<0.024	30.110	21.800	<0.027	1.720	65.060	3.786	25.239	5.915
378o	38.400	6.110	2.710	<0.023	30.210	21.740	<0.027	1.680	65.267	3.716	23.817	7.200
378p	38.400	7.580	1.960	<0.023	29.000	21.810	<0.027	1.700	61.826	3.778	29.654	4.743

Grain number	electron microprobe data [wt%]								normalised endmembers calculated[59] [mol%]			
	SiO_2	MgO	CaO	TiO_2	FeO_{total}	Al_2O_3	Cr_2O_3	MnO	Alm	Sps	Prp	Grs
378q	38.710	6.880	2.200	<0.023	29.890	22.000	<0.027	1.680	63.849	3.682	26.542	5.926
378r	38.530	6.330	2.870	<0.023	29.240	21.670	<0.027	1.650	63.826	3.649	24.634	7.891
378s	38.510	7.510	1.550	<0.023	29.680	21.890	<0.027	1.680	63.271	3.716	29.235	3.777
378t	38.020	7.880	1.510	<0.023	29.380	21.680	<0.027	1.710	61.975	3.866	31.374	2.784
379	38.400	6.820	3.700	<0.024	28.110	21.870	<0.027	1.640	59.880	3.636	26.608	9.875
380	38.310	4.130	6.850	<0.023	28.320	21.980	0.040	1.400	61.752	3.098	16.092	19.059
381	39.680	8.790	8.750	0.072	20.810	22.230	<0.026	0.331	43.362	0.722	33.482	22.433
382	38.620	7.890	1.275	<0.024	30.130	22.160	0.053	0.870	64.199	1.909	30.509	3.383
383	38.280	5.330	3.890	0.033	30.010	21.810	<0.027	1.470	65.187	3.253	20.765	10.794
384	39.230	9.230	2.050	<0.023	27.740	22.130	<0.027	0.911	58.042	1.985	35.495	4.479
385	38.640	8.410	1.700	<0.023	29.030	22.080	<0.026	0.653	61.508	1.433	32.495	4.563
386	39.650	10.980	1.560	<0.023	25.390	22.490	0.041	0.813	52.936	1.742	41.414	3.908
387	39.230	6.750	9.920	0.068	22.350	22.230	<0.026	0.381	46.863	0.830	25.815	26.493
388	39.020	5.600	11.940	0.029	20.810	22.060	<0.026	1.024	43.924	2.243	21.527	32.306
389	38.830	5.730	11.740	0.064	21.060	22.170	0.051	0.436	44.875	0.958	22.044	32.124
390a	38.860	5.670	8.000	0.051	25.850	22.110	<0.027	0.501	55.222	1.095	21.761	21.922
390b	38.300	6.580	6.750	0.034	25.750	21.760	0.027	0.783	54.485	1.749	25.918	17.848
391	38.930	7.120	5.040	<0.022	26.010	22.010	<0.027	1.400	55.849	3.052	27.279	13.820
392	38.720	5.920	9.390	<0.023	23.730	21.610	<0.027	0.832	50.353	1.851	23.228	24.568
393	38.880	7.030	5.260	<0.023	27.290	21.900	<0.026	0.417	58.133	0.920	27.190	13.757
394	38.670	6.670	6.040	0.024	25.670	21.630	<0.027	1.540	55.031	3.429	26.096	15.444
396	38.360	7.090	2.330	<0.024	29.590	21.960	<0.027	1.190	63.308	2.629	27.554	6.508
397	40.620	13.650	3.720	0.040	19.590	22.950	<0.026	0.351	39.699	0.738	50.429	9.134
398	38.530	7.290	5.420	<0.023	25.410	21.870	<0.026	1.290	54.159	2.850	28.348	14.644
399	38.720	5.910	10.680	0.034	22.240	22.100	<0.027	0.597	46.633	1.316	22.846	29.205
400	38.500	0.443	22.400	0.098	17.650	21.190	<0.026	0.269	36.822	0.614	1.781	60.783
452a	40.070	5.660	6.850	<0.023	25.540	22.560	<0.027	0.923	56.329	2.058	22.254	19.359
452b	39.190	6.190	6.260	<0.024	26.550	21.990	<0.026	0.965	57.117	2.098	23.740	17.045
452c	38.870	6.040	6.370	0.030	26.100	21.970	<0.026	0.959	56.739	2.114	23.402	17.745
452d	38.660	6.220	7.270	0.034	25.760	21.440	<0.026	0.833	55.337	1.864	24.524	18.275
452e	37.780	6.370	7.110	<0.023	25.720	21.200	<0.026	0.871	54.288	2.014	25.876	17.821
452f	36.670	6.840	6.850	0.031	25.400	20.520	<0.027	0.836	52.977	2.032	29.277	15.714
452g	37.250	6.570	6.580	<0.022	25.560	20.990	<0.027	0.889	54.299	2.082	27.061	16.559

Grain number	electron microprobe data [wt%]								normalised endmembers calculated[59] [mol%]			
	SiO_2	MgO	CaO	TiO_2	FeO_{total}	Al_2O_3	Cr_2O_3	MnO	Alm	Sps	Prp	Grs
452h	38.110	6.180	7.030	0.045	25.760	21.290	<0.027	0.839	55.474	1.902	24.704	17.919
452i	38.660	6.060	7.230	0.051	26.050	21.860	<0.026	0.852	55.383	1.881	23.605	19.132
452j	38.330	6.180	7.500	0.031	25.480	21.700	<0.027	0.764	54.062	1.707	24.343	19.888
452k	38.640	6.110	7.370	0.029	25.860	21.990	<0.026	0.835	54.579	1.848	23.726	19.848
452l	39.070	6.320	6.330	<0.023	26.360	22.040	<0.027	0.908	56.581	1.973	24.179	17.268
452m	38.770	6.110	7.240	<0.023	25.460	21.890	<0.026	0.801	54.866	1.754	23.544	19.836
452n	38.120	6.000	7.710	0.037	25.310	21.580	<0.027	0.736	53.997	1.656	23.768	20.579
706	38.960	7.430	5.530	<0.023	25.920	22.040	<0.026	0.819	54.904	1.788	28.603	14.706
707	38.290	7.550	0.993	<0.023	30.150	21.840	<0.027	1.850	64.165	4.118	29.591	2.125
708	38.390	7.440	1.211	<0.023	30.140	21.960	<0.027	1.470	64.484	3.243	28.888	3.384
709	40.360	14.120	5.120	0.027	16.860	23.130	0.048	0.545	33.256	1.145	52.301	13.298
710	38.430	6.520	6.600	<0.023	25.990	21.940	<0.026	0.450	55.379	0.998	25.325	18.298
711	38.760	6.170	8.450	0.029	24.850	22.090	<0.026	0.520	52.142	1.147	23.873	22.838
712	38.020	4.880	5.220	<0.023	25.810	21.690	<0.027	4.920	55.393	10.998	19.203	14.407
713	38.190	8.000	1.960	0.023	28.910	21.990	<0.027	1.244	60.853	2.774	31.375	4.998
714	38.460	7.650	1.177	<0.023	29.310	21.770	<0.026	1.560	63.645	3.432	29.661	3.262
715	38.070	6.860	1.398	<0.023	31.120	21.840	<0.027	1.139	66.714	2.536	26.880	3.870
716	38.390	8.430	2.190	<0.023	27.900	21.950	<0.026	1.063	59.065	2.354	32.880	5.702
717	37.800	3.860	10.010	<0.023	25.450	21.130	<0.027	1.610	54.268	3.707	15.649	26.376
718	38.440	8.230	1.175	<0.023	29.380	21.950	<0.026	1.010	62.688	2.230	31.964	3.118
719	38.810	8.330	8.840	0.066	21.400	21.860	<0.026	0.316	43.975	0.703	32.699	22.623
720	38.500	5.660	10.440	0.042	23.030	21.810	<0.026	0.483	48.593	1.071	22.160	28.177
721	38.320	6.480	7.350	0.038	25.630	21.840	<0.026	0.464	53.885	1.034	25.489	19.593
722	37.840	6.490	1.061	<0.023	29.780	21.830	<0.027	3.130	64.421	7.011	25.568	3.000
723	38.030	9.540	1.450	<0.022	27.590	21.700	<0.027	0.770	57.741	1.747	38.012	2.501
724	38.000	4.590	7.890	0.031	27.390	21.530	<0.026	0.552	59.124	1.240	18.176	21.460
725	39.070	8.650	9.990	0.058	18.920	22.250	0.045	0.354	39.190	0.771	33.212	26.827
726	38.110	5.560	1.430	<0.024	31.760	21.630	<0.026	2.300	69.201	5.122	21.771	3.906
727	39.690	11.440	6.270	0.035	19.730	22.490	<0.026	0.357	39.882	0.776	43.476	15.867
728	38.900	8.160	4.250	0.027	26.510	21.760	<0.026	0.937	56.072	2.079	31.866	9.984
729	38.420	7.290	1.790	<0.023	28.890	21.970	<0.027	2.180	61.910	4.810	28.288	4.991
730	38.230	4.950	7.220	0.033	27.730	21.640	<0.027	0.517	59.826	1.155	19.449	19.571
731	38.270	5.910	8.480	0.024	24.600	21.550	<0.026	0.610	52.652	1.367	23.337	22.644

Grain number	electron microprobe data [wt%]								normalised endmembers calculated[59] [mol%]			
	SiO_2	MgO	CaO	TiO_2	FeO_{total}	Al_2O_3	Cr_2O_3	MnO	Alm	Sps	Prp	Grs
733	38.000	6.320	2.680	<0.023	30.030	21.720	<0.026	1.167	65.053	2.597	24.791	7.559
734	38.450	4.630	8.620	0.064	26.310	21.580	<0.027	0.986	56.739	2.197	18.198	22.866
735	38.330	7.690	5.380	<0.022	25.360	21.700	<0.026	1.142	53.341	2.566	30.371	13.723
736	38.840	7.290	6.040	<0.023	26.130	22.050	<0.026	0.559	54.666	1.233	28.250	15.852
737	38.500	8.520	1.036	<0.023	29.530	22.000	<0.026	0.638	62.827	1.403	33.044	2.726
738	39.730	12.180	6.920	<0.022	17.810	22.690	<0.026	0.405	35.102	0.869	46.131	17.898
739	37.460	1.850	9.290	0.047	29.480	21.290	<0.027	1.490	63.749	3.426	7.479	25.347
740	37.370	2.370	8.440	0.034	19.970	21.160	<0.027	10.480	43.156	24.031	9.568	23.246
741	40.370	12.990	5.280	0.026	18.750	22.920	<0.026	0.347	37.591	0.730	48.297	13.382
742	38.440	7.290	3.110	<0.022	28.980	21.950	<0.027	0.745	61.621	1.649	28.362	8.368
743	38.580	8.040	1.670	<0.023	28.900	22.060	<0.026	1.082	61.921	2.373	31.071	4.635
744	38.580	5.520	9.940	0.043	24.320	21.410	<0.026	0.438	51.576	0.992	21.924	25.509
745	37.510	6.370	1.750	<0.023	31.070	21.440	<0.026	1.550	66.913	3.537	25.592	3.958
746	38.820	6.110	8.770	<0.023	23.640	22.190	<0.026	0.829	50.517	1.807	23.466	24.209
747	38.550	8.830	1.104	<0.023	28.550	22.260	0.028	0.869	60.932	1.909	34.179	2.980
748	37.940	7.060	1.830	0.026	29.930	21.880	<0.027	1.720	63.519	3.851	27.832	4.798
749	38.590	7.810	4.280	0.025	27.070	21.890	<0.026	0.688	57.118	1.531	30.503	10.848
750	38.810	8.910	6.390	<0.022	22.450	22.040	<0.026	0.763	46.854	1.677	34.513	16.956
751	38.510	4.250	13.040	0.028	21.390	21.750	<0.026	0.737	45.961	1.634	16.551	35.855
752	38.320	7.240	1.490	<0.023	29.910	21.850	<0.026	1.400	64.571	3.094	28.167	4.169
753	38.600	7.100	10.060	0.026	21.360	21.960	<0.026	0.482	44.169	1.065	27.746	27.020
754	38.470	9.160	1.490	<0.023	27.980	22.020	<0.027	0.622	59.155	1.377	35.599	3.869
755	38.880	9.710	1.306	<0.023	26.200	22.060	<0.027	1.770	55.532	3.873	37.378	3.217

[59]Locock, A. J. An Excel spreadsheet to recast analyses of garnet into end-member components, and a synopsis of the crystal chemistry of natural silicate garnets. *Comput. Geosci.* **34,** 1769-1780 (2008).
